# Interleukin-1 and estrogen protect against disseminating dentoalveolar infections

**DOI:** 10.1038/ijos.2016.61

**Published:** 2017-03-30

**Authors:** Hesham Youssef, Philip Stashenko

**Affiliations:** 1 Host-Microbiome Center, Department of Applied Oral Sciences, The Forsyth Institute, Cambridge, USA

**Keywords:** cytokines, dentoalveolar, disseminating infections, estrogen, neutrophils

## Abstract

Dentoalveolar bacterial infections cause localized tissue and bone destruction, but usually remain well-localized within teeth in immunocompetent hosts. However, in certain cases these infections may invade head and neck tissues, resulting in orofacial abscesses, cellulitis and sepsis, with resultant high morbidity and even mortality. In the present studies, we developed a novel model of spreading dentoalveolar infections in mice by treatment with neutralizing antibodies against both interleukin-1α (IL-1α) and IL-1β. Surprisingly male but not female mice given anti-IL-1 antibodies developed orofacial abscesses, weight loss, splenomegaly and sepsis. Female mice developed abscesses and sepsis comparable to males following ovariectomy (OVX), which was reversed by estrogen supplementation. Anti-IL-1 blockade inhibited IL-12, interferon γ (IFNγ) and IL-6 but not IL-10 expression in infrabony lesions, suggestive of a local anti-inflammatory response. There was greater infiltration of neutrophils and other inflammatory cells into lesions in anti-IL-1-treated animals; however, blood leukocytes had reduced bacterial phagocytic and killing activity *ex vivo*. Estrogen directly stimulated IL-1 production by macrophages, suggesting that the resistance of females to disseminating dentoalveolar infections may be due to their heightened pro-inflammatory responses following bacterial challenge, leading to enhanced localization of these infections.

## Introduction

Dentoalveolar abscesses and disseminating infections of endodontic origin occur as sequellae of bacterial invasion of the dental pulp. Dentoalveolar abscesses result from the large-scale egress of bacteria from the root canal system into surrounding tissues, which evade ingestion and destruction by phagocytic leukocytes and other local immune mechanisms. If not promptly and effectively treated, these infections can rapidly spread by dissection along fascial planes, and result in cellulitis, sinusitis, deep space infections of the head and neck, intracranial abscesses and frank sepsis, resulting in significant morbidity and even mortality.^1-2^

The immune responses that localize these infections and prevent their dissemination are not fully understood. In previous studies, we showed that B-cell- but not T-cell-deficient mice were susceptible to abscess formation and sepsis, and that passively transferred antibody, primarily IgG, against infecting bacteria was protective.^3-4^ Mice doubly deficient in P- and E-selectin and reduced phagocyte migration, had decreased ability to slow the progress of dentoalveolar infections.^5^ Conversely, animals treated with the immunomodulator poly-(1-6)-beta-D-glucopyranosyl-(1-3)-beta-D-glucopyranose (PGG)-glucan, which enhances neutrophil production and priming, had greater infection resistance.^6^ Taken together these data indicate that antibody-mediated bacterial opsonization, combined with efficient phagocytosis and killing of bacteria by neutrophils are critical elements for protection.

In the present study, we further investigated the role of phagocytic leukocyte priming in the context of sex specificity, using a novel mouse model of disseminating dentoalveolar infections.

## Materials and methods

### Mice

Eight-week-old C57Bl/6 male and female mice were purchased from Jackson Laboratory, Bar Harbor, ME, USA. Ovariectomized (OVX) and sham OVX 9-week-old C57Bl/6 female mice were obtained from Jackson Laboratory 2 weeks after surgery. Animals were maintained in a conventional environment in the AAALAC accredited Forsyth Institute Animal Facility, according to the guidelines of the Institutional Animal Care and Use Committee (IACUC) under protocol #14-009. All procedures conformed to the National Institutes of Health (NIH) guide for the care and use of laboratory animals (NIH publication no. 8023, 2010).

### Dentoalveolar infections

For infection induction, mice were mounted on a jaw retraction board and anesthetized with ketamine HCI (62.5 mg·kg^−1^) and xylazine (12.5 mg·kg^−1^) in sterile phosphate-buffered saline by intraperitoneal injection. Both lower first molar dental pulps were exposed using a #1/4 round bur under a surgical microscope (MC-M92; Seiler, St Louis, MO, USA) as described.^7^ Animals without infection served as negative controls. Four human dentoalveolar pathogens, *Prevotella intermedia* ATCC 25611, *Streptococcus intermedius* ATCC 27335, *Fusobacterium nucleatum* ATCC 25586 and *Peptostreptococcus micros* ATCC 33270 were grown on tryptic soy broth with yeast agar plates under anaerobic conditions (80% N_2_, 10% H_2_, 10% CO_2_), harvested and cultured in Mycoplasma liquid media (PPLO, Fisher Scientific, Pittsburgh, PA, USA). The cells were centrifuged and resuspended in pre-reduced anaerobically sterilized Ringer's solution (PRAS; Anaerobe Systems, Morgan Hill, CA, USA). Equal numbers of the four bacteria were mixed, placed into 2g of methylcellulose per mL; 4 × 10^8^ total bacteria were transferred into the tooth pulp chamber and teeth were sealed with CAVIT.

### Antibodies and neutralization

Goat anti-mouse interleukin (IL)-1α and IL-1β affinity-purified polyclonal antibodies (IgG) were purchased from R&D Systems, Minneapolis, MN, USA. Mice received 10 μg antibodies subcutaneously on days 0, 3, 6, 9 and 12 relative to infection. Control mice received control goat IgG on the same schedule. On day 21 all mice were killed and samples prepared as described below.

### Estrogen (E2) supplementation

Tablets of 17β-estradiol (0.5 mg, 21 day slow release; Innovative Research of America, Sarasota, FL, USA) were surgically implanted subcutaneously in the backs of mice on day 0. 17β-estradiol (Sigma, St Louis, MO, USA) was also used in cell cultures to determine effects on bacteria stimulated IL-1 production.

### Sample preparation

Animals were killed by CO_2_ inhalation, and mandibles were removed and infrabony tissues from the right hemi-mandible isolated under a dissecting microscope. Tissues were weighed and placed into 1 mL of lysis buffer and ground using a sterile tissue homogenizer as described.^8^ The mixture was incubated at 4 °C for 1 h, and the supernatant was collected after centrifugation and stored at −80 °C until assay. Left hemi-mandibles were fixed in 4% paraformaldehyde, decalcified, embedded in paraffin, sectioned at 7 μm and stained with hematoxylin and eosin. The number of neutrophils and total inflammatory cells were counted under high power (× 200) within three predetermined fields including the right, center and left of the infrabony lesions, and means±standard deviation calculated.

### Macrophage cultures

Unstimulated macrophages were collected by lavage with 3 mL cold phosphate-buffered saline (PBS) from the peritoneal cavity of normal C57Bl/6 female mice, and cultured in medium containing estrogen-depleted fetal bovine serum (FBS) (Invitrogen, Carlsbad, CA, USA) in the absence/presence of 17β-estradiol (estrogen, E2; Sigma, St Louis, MO, USA) at log_10_ dilutions (1 × 10^−4^–1 pg·mL^−1^) and/or *E. coli* lipopolysaccharide (LPS) (100 ng·mL^−1^) in triplicate. Supernatants were collected after 24 h and assayed for IL-1α and IL-1β by enzyme-linked immunosorbent assay (ELISA).

### Cytokine assays

ELISAs for cytokines in tissue extracts employed commercially available kits for mouse IL-1α, IL-1β, IL-4, IL-6, IL-10, IL-12, interferon γ (IFNγ) and TNFα (all from BioSource International, Camarillo, CA, USA). All assays were carried out according to the manufacturer's instructions. Results were expressed as pg cytokine/mg infrabony tissue.

### Bacterial phagocytosis and killing assays

Assays were performed to measure the phagocytosis and killing of *Fusobacterium nucleatum (F. nucleatum)* as a representative pathogen, as described^9^ using *ex vivo* leukocytes. In brief, whole blood (1 mL) was collected by cardiac puncture at killing on day 21 into a heparinized syringe, added to 9 mL dextran and allowed to sediment for 1h. The leukocyte rich buffy coat was collected, cells were counted, mixed with an equal volume of freshly grown *F. nucleatum* (10^6^ cells per mL in PRAS medium), and incubated at 37 °C with rotation. Duplicate samples were taken at 0, 10, 20 and 30 min, and added to ice-cold PBS to stop the assay. The samples were centrifuged and the supernatants plated on pre-marked areas on blood agar plates (two cultures /sample/time point) to assess the numbers of non-internalized bacteria. To measure internalized bacteria, the leukocyte pellet was washed in ice-cold PBS, homogenized with 20 μL Saponin (Sigma, St Louis, MO, USA) in 1 mL ice-cold PBS, and the cell contents plated on blood agar plates. Plates were cultured for 3 days under anaerobic conditions, and bacterial colonies were counted under a dissecting microscope.

### Statistical analysis

Student's *t*-test was used to analyze the effect of E2 and lipopolysaccharide (LPS) on IL-1α and IL-1β production. One-way analysis of variance (ANOVA) with Tukey's standardized test was used for neutrophil and total inflammatory cell counts, animal and spleen weights, and the levels of cytokines. A mixed model repeated measures analysis of group/time effects was used to analyze phagocytosis and killing assays. *χ*^2^ with the Bonferroni correction was used for frequency of abscess formation.

## Results

### Effect of systemic neutralization of IL-1α and IL-1β on abscess formation and sepsis

**Figure 1:**
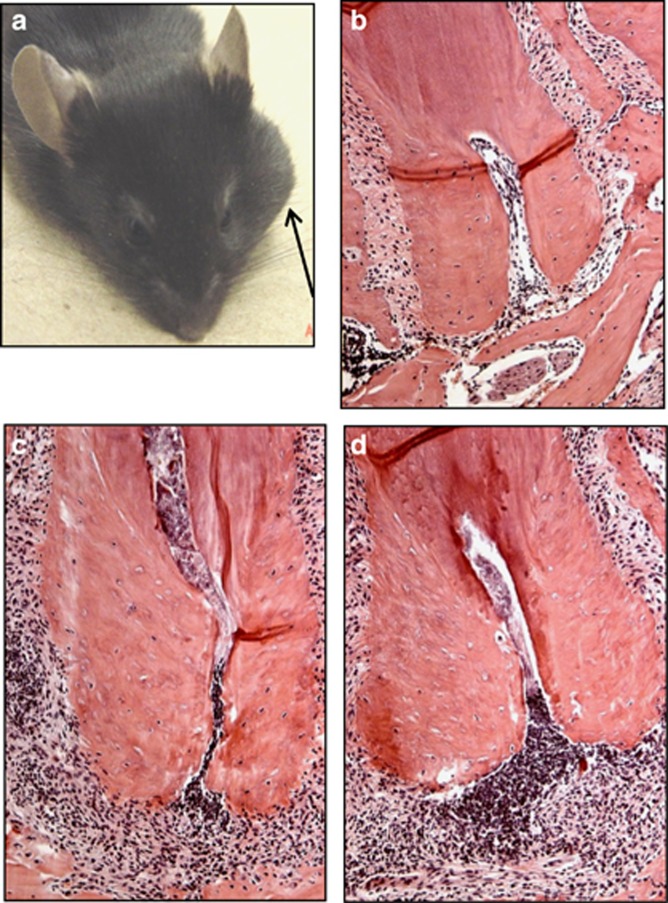
**Pathology of infrabony lesions after dentoalveolar infection.** (**a**) Male mouse subjected to infection and treatment with anti-IL-1α/β neutralizing antibodies *in vivo*, demonstrating a large orofacial abscess (arrow). Histology of: (**b**) uninfected control; (**c**) infected dental pulp, mouse treated with unreactive IgG; (**d**) infected, anti-IL-1α/β antibody. Note normal cellular detail, close apposition of bone to tooth in uninfected dental pulp in (**b**) indicating viable tissue, and absence of infiltrating inflammatory cells in the infrabony area surrounding the tooth. Cellular detail is lost in (**c**, **d**) indicative of dental pulp necrosis as a result of infection. The greatest number of infiltrating cells was present in lesions in mice that received anti-IL-1α/β antisera (panel **d**; see also Figure 2). Paraffin sections, hematoxylin and eosin staining; magnification × 200. IgG, immunoglobulin G; IL, interleukin.

We determined the effect of the functional inhibition of IL-1α and/or IL-1β on the resistance of mice to disseminating infections, by blocking IL-1 isoforms with neutralizing antibodies. Mice were subjected to dentoalveolar infection with a mixture of four common human endodontic pathogens, and were treated subcutaneously with neutralizing antibodies against mouse IL-1α, IL-1β, or both (anti-IL-1α/β) on days 0, 3, 6, 9 and 12 relative to infection, or received unreactive goat IgG as a control. As seen in Table 1, both male and female mice treated with either anti-IL-1α or anti-IL-1β alone as well as controls treated with unreactive IgG did not develop abscesses or sepsis. In contrast, most (6/8) male mice treated with the combination of anti-IL-1α plus anti-IL-1β antibodies (anti-IL-1α/β) developed orofacial abscesses beginning 5–7 days after infection, pathognomonic for disseminating dentoalveolar infections (Figure 1a). Abscesses manifested as large swellings that extended into facial spaces. Affected animals also had reduced physical activity, loss of body weight and splenomegaly, all indicators of sepsis. In contrast only 1 of 8 female mice treated with the anti-IL-1α/β combination developed a small abscess, but did not exhibit weight loss or splenomegaly, indicating a localized lesion. These data suggest that the functional absence of both IL-1 isoforms, which signal through the common IL-1RI, results in an inability of male mice to localize dentoalveolar infections, leading to abscesses and sepsis.

### Cell Infiltration in anti-IL-1-treated mice

**Figure 2:**
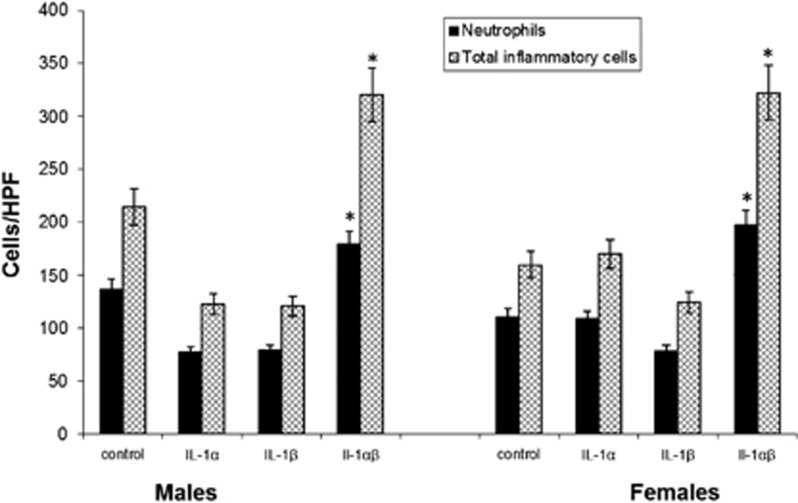
**Infrabony inflammatory cell infiltration in anti-IL-1-treated male and female mice subjected to dentoalveolar infection.** The mean number of total inflammatory cells and neutrophils was determined in three high-powered fields in each treatment group (*n*=8). A significant increase in infiltrating neutrophils and other inflammatory cells occurred in mice treated with anti-IL-1α/β antisera *vs* all other groups. There were no differences between males and females. Vertical bars: standard deviation *P=0.05 by ANOVA. ANOVA, one-way analysis of variance; HPF, high-powered field; IL, interleukin.

Mandibles from each animal were processed for histology (Figure 1, panels **b**–**d**), and infiltrating neutrophils and total inflammatory cells were quantified (Figure 2). Animals receiving either antibody alone showed slightly reduced cell infiltration (not significant (NS) *vs* controls), whereas those treated with the anti-IL-1α/β combination had the greatest number of infiltrating neutrophils and total inflammatory cells after 21 days (Figure 2, *P*<0.03). This indicates that neutrophil migration was not affected by anti-IL-1α/β blockade, suggesting that another function may have been compromised leading to a failure to localize infections.

### Cytokine expression in infrabony lesions

**Figure 3:**
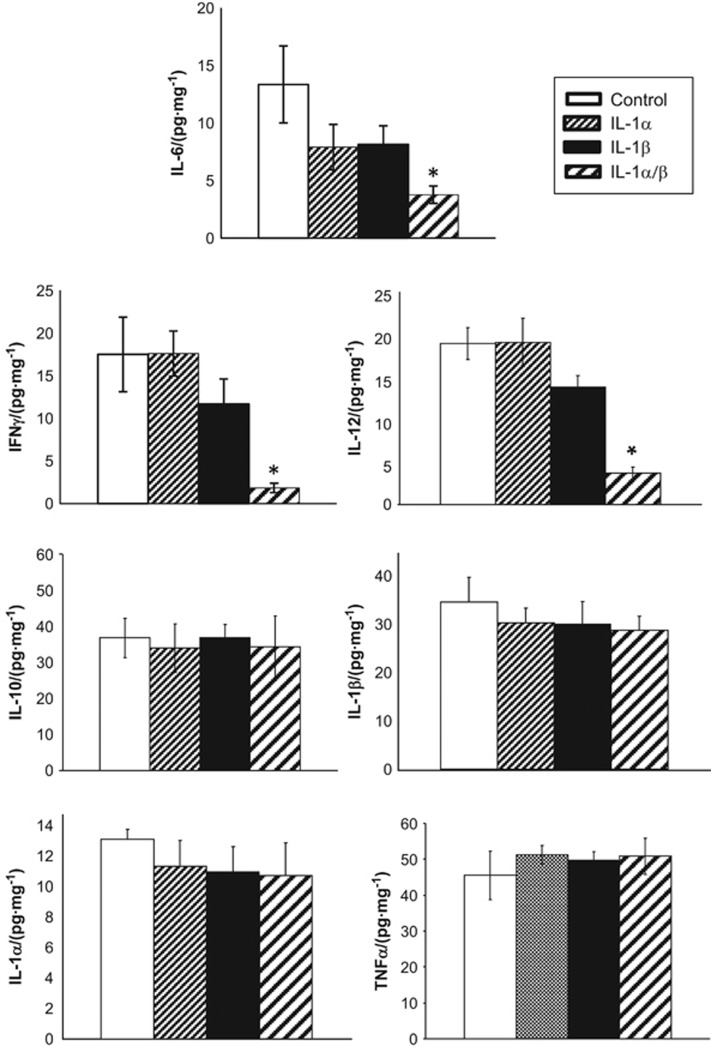
**Effect of anti-IL-1 neutralizing antibodies on cytokine expression in infrabony lesions; pooled data from male and female mice.** Significant reductions were seen in tissue levels of IL-12, IFNγ and IL-6 in mice treated with anti-IL-1α/β antibodies; all other cytokines were unchanged (*n*=16 animals/group). Bars and vertical lines: means±standard deviation *P<0.01 *vs* control IgG by ANOVA. ANOVA, one-way analysis of variance; IFNγ, interferon-γ IL, interleukin; TNFα, tumour necrosis factor α.

**Figure 4:**
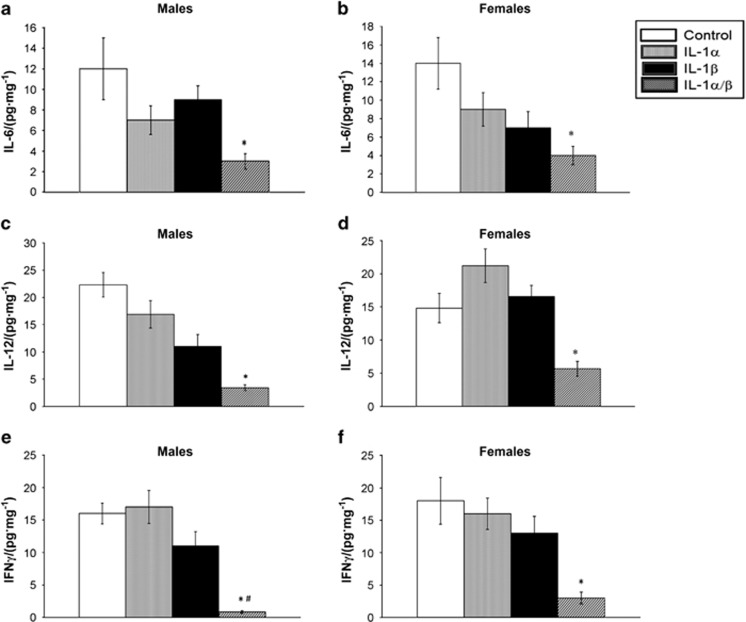
**Effect of anti-IL-1 antibodies on cytokine expression in infrabony lesions in male *vs* female mice.** Panels: infrabony lesion tissue levels of: (**a**) IL-6, males; (**b**) IL-6, females; (**c**) IL-12, males; (**d**) IL-12, females; (**e**) IFNγ, males; (**f**) IFNγ, females. *P<0.01 *vs* control IgG treatment by ANOVA. A significantly greater reduction in IFNγ occurred in males *vs* females treated with anti-IL-1α/β antibodies. ^#^*P*<0.05 *vs* females by *t*-test. *n*=8 animals/group; bars and vertical lines: means±standard deviation ANOVA, one-way analysis of variance; IFNγ, interferon-γ IgG, immunoglobulin G; IL, interleukin; TNFα, tumour necrosis factor α.

The levels of cytokines in infrabony lesions were evaluated by ELISA on day 21 post-infection (Figure 3). Both male and female mice treated with the combination of anti-IL-1α/β antibodies showed a significant suppression of IL-6, IL-12 and IFNγ compared with controls and to mice treated with either antibody alone, indicating that IL-1RI signaling positively regulates all three mediators. In contrast, IL-10, IL-1α, IL-1β and tumour necrosis factor α (TNFα) showed no differences among treatment groups. As shown in Figure 4, tissue levels of IFNγ were significantly lower in males than in females, and IL-12 and IL-6 trended lower but were not significant. Taken together these findings suggest the induction of a more predominant anti-inflammatory periapical environment in males *vs* females.

### Effect of estrogen on infection dissemination

The increased susceptibility of males *vs* females to infection dissemination following IL-1 blockade suggested that sex-related factors, in particular estrogen, could be responsible for this difference. To determine the role of estrogen, female mice were OVX or treated by sham surgery as a control (SHAM). OVX females were further separated into two groups, one of which received a slow release estrogen implant subcutaneously that provided estrogen over 21 days, and the other without supplementation. The animals were then either given injections of anti-IL-1α/β antibody or unreactive IgG as a control as above.

**Figure 5:**
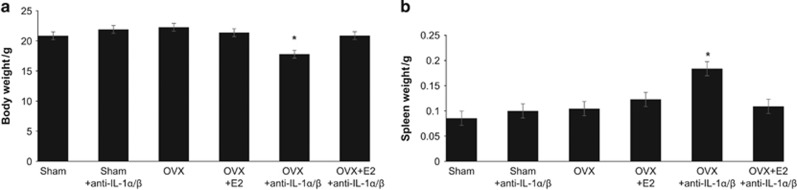
**Effect of anti-IL-1α/β antibodies and estrogen modulation on body weight and splenomegaly.** (**a**) Body weight was significantly reduced and (**b**) spleens were enlarged in mice lacking estrogen that also received anti-IL-1α/β antibodies (*n*=11 animals/group). Bars and vertical lines: mean±standard deviation *P<0.05 *vs* controls by ANOVA. ANOVA, one-way analysis of variance; IL, interleukin.

As seen in Table 2, OVX female mice that also received anti- IL-1α/β antibodies developed a high frequency of abscess development (7/10) and signs of infection dissemination including weight loss (Figure 5a, *P*<0.04) and splenomegaly compared with controls (Figure 5b, *P*<0.04). Infection dissemination was nearly completely reversed by supplementation with estrogen (17β-estradiol; Table 2) as were weight loss and splenomegaly. None of the other groups developed significant numbers of abscesses, again confirming the role of anti-IL-1α/β blockade in this outcome.

### *Ex vivo* phagocytosis and killing studies

**Figure 6:**
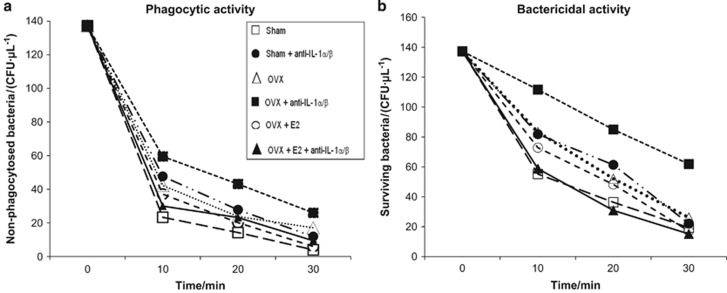
**Effect of anti-IL-1 antisera and OVX on phagocytosis and killing of bacteria by peripheral blood neutrophils.** Panels: (**a**) more bacteria remained non-internalized, and (**b**) fewer internalized bacteria were killed in the OVX+anti-IL-1α/β treated group *vs* all other groups (*n*=11). *P<0.004, multiple regression analysis. IL, interleukin; OVX, ovariectomy.

As seen above (Figure 2), the increased numbers of infiltrating leukocytes in infrabony lesions of anti-IL-1α/β-treated animals indicated that cell migration was not affected, and that infection dissemination may be explained by reduced phagocytic cell bactericidal activity. To investigate this possibility, the phagocytic and bactericidal activities of peripheral blood leukocytes isolated from animals in the preceding OVX experiment were tested using *F. nucleatum* as a model pathogen. As seen in Figure 6a, blood leukocytes isolated from OVX mice that received anti-IL-1α/β had the lowest phagocytic activity (solid squares, highest number of non-phagocytosed *F. nucleatum*) at all times tested. This effect was reversed in leukocytes from animals subjected to OVX and treated with anti-IL-1α/β but also given estrogen supplementation (*P*<0.004). The latter mice as well as leukocytes from OVX+E2 treated mice had higher phagocytic activity that approached that of the negative control (sham OVX, no anti-IL-1α/β). A similar result was obtained when the killing of *F. nucleatum* was determined (Figure 6b). The poorest killing of internalized bacteria was observed in the anti-IL-1α/β+OVX group (*P*<0.004); killing was increased and approached control levels with estrogen supplementation.

### IL-1 production by macrophages in response to LPS and estrogen

**Figure 7:**
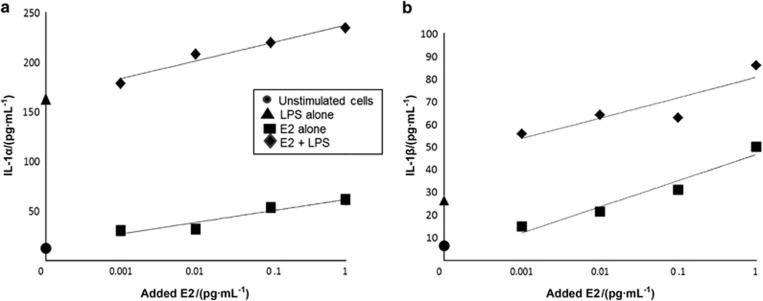
**Effect of estrogen (E2) and LPS on IL-1α and IL-1β production by macrophages *in vitro*.** The effect of E2 and LPS on both IL-1 isoforms was additive (*P<0.05, *t*-test). E2, estrogen; IL, interleukin.

To determine if estrogen upregulates IL-1 as a possible mechanism for increased protection against disseminating infections, we assessed the effect of 17β-estradiol on the production of IL-1 by macrophages. Unstimulated macrophages were isolated from the peritoneal cavities of female C57BL6 mice, and were cultured in estrogen-free medium in the presence/absence of LPS and estrogen. As expected, LPS stimulated both IL-1α and IL-1β expression (Figure 7). However, estrogen alone also increased production of both IL-1α and IL-1β in a dose-dependent manner, even at very low concentrations (0.01 μg·mL^−1^). When cells were stimulated with the combination of LPS and estrogen, an additive rather than a synergistic response was observed. These data suggest that females, by the virtue of their estrogen production, may be relatively resistant to dentoalveolar infection dissemination because they produce higher constitutive levels of IL-1, resulting in greater neutrophil priming than males.

## Discussion

Spreading dentoalveolar infections constitute a significant and growing public health problem, and in severe cases are life-threatening. In the present study, we undertook to further characterize the host immune factors that protect against disseminating dentoalveolar infections in a novel mouse model. Our results indicate that the priming and activation of neutrophils at least in part by estrogen, possibly via increased expression of IL-1, comprises a critical host defense mechanism.

There is compelling evidence from human epidemiological studies that females are more resistant to infectious challenges and sepsis than males, whereas females are more susceptible to autoimmune diseases.^10, 11, 12, 13^ Post-trauma mortality from sepsis was also higher in males younger than 50 years of age, a difference that disappeared in patients older than 50 years, suggestive of menopausal changes in females.^14^ Along these lines pre-pubertal females and males had similar sepsis mortality, but this was reduced by ~50% after puberty in females.^15^

Circulating estrogen levels are most closely correlated with infection resistance, and estrogen has been shown to regulate the expression of a broad array of sexually dimorphic autosomal genes in human peripheral blood cells.^16^ Female-biased gene ontology categories were highly enriched for various immune system functions, including TLR3 and TLR4 pathways (poly I:C and LPS responses), genes linked to the autoimmune diseases, as well as genes regulated by estrogen and LPS. Significant subcategories included responses to cytokine and type 1 interferon stimulation, and lymphocyte differentiation. These functional expression patterns are also reflected in sexually dimorphic immune responses in mice,^17^ leading to increased humoral,^18^ T cell^19^ and innate immune responses.^20^ As noted above, B-cell-deficient mice are susceptible to dentoalveolar sepsis, but are largely protected by the passive transfer of anti-bacterial IgG antibodies. As C5 complement-deficient mice were not susceptible, it is likely that opsonization of bacteria to enhance phagocytosis by neutrophils and macrophages is a key protective mechanism.^3-4^ CD4+ T cells may also participate, principally by providing ‘help' for B cells.^19^

In contrast male hormones are reported to be immunosuppressive,^21^ and treatment with the androgen receptor antagonist flutamide increased cytokine responses in male mice with trauma and sepsis, and improved survival.^22-23^ Treatment with the estrogen receptor-beta agonist WAY-202196 improved survival, preserved intestinal epithelial integrity, and significantly reduced systemic bacteremia and peritoneal interleukin-6 and tumor necrosis factor levels in the same model, and provided a comparable level of protection in both male and female animals^24^ Taken together these studies indicate that although estrogen may protect primarily by enhancing neutrophil priming and bactericidal activity, and indirectly through stimulation of antibody responses, other estrogen-stimulated mechanisms could also be operative.

Sex differences in disseminating infections of dentoalveolar origin have been less clear. More males than females were hospitalized with severe dentoalveolar infections in several studies,^25-26^ whereas equal frequencies were reported in others.^27-28^ In a recent large retrospective analysis of more than 60 000 cases from 2000–2008, more females than males were hospitalized for severe periapical abscesses.^29^ However of 66 deaths in this cohort, 42 were males and 24 were females (*P*<0.02; V. Allareddy, personal communication). A similar mortality bias was observed in a study of more than four million emergency room visits from 2008 to 2010 for conditions including pulpal/periapical lesions, oral cellulitis or abscess.^30^ Of 101 deaths in this series, 58 were males and 43 were females (V. Allareddy, personal communication). It thus appears that sepsis of dentoalveolar origin also has more severe effects on males than females, similar to other body sites, and consistent with the findings in the present study. A similar gender-biased mortality as observed in the present study was also reported using IL-1 and TNF receptor knockout mice,^31^ albeit the cellular and molecular mechanisms underlying resistance in females were not determined.

A question remains whether the effects of IL-1 on phagocytic leukocytes is direct, or indirect via induction of other pro-inflammatory mediators such as IL-12 and IFNγ, both of which have been reported to protect against polymicrobial sepsis.^32^ IL-1 can directly prime neutrophils and monocytes for enhanced anti-microbial activity.^33, 34, 35^ However in our studies, IL-1α, IL-1β and TNFα levels were unchanged following anti-IL-1 blockade (Figure 3), suggesting an indirect effect. In contrast, periapical lesion levels of IL-6, IL-12 and IFNγ were significantly reduced and IL-10 was unchanged, suggestive of the induction of a predominantly anti-inflammatory local immune response. Of note, IFNγ was significantly lower in males *vs* females and IL-12 trended lower, suggesting that inhibition of these cytokines may have a critical role in male susceptibility. Further studies are required to resolve this issue.

This local anti-inflammatory mileau is furthermore consistent with a predominance of N2 neutrophil and M2 macrophage infiltration.^36^ The recent description of distinct neutrophil phenotypic/functional subsets^37^ may be relevant. First described in the context of solid tumors similar to polarized macrophages, neutrophils may exhibit either a pro-inflammatory (N1) or anti-inflammatory/immunosuppressive (N2) functional profile, with the production of immunosuppressive cytokines including IL-10, and TGFβ.^38, 39, 40, 41^ For example in sepsis following burn injury, circulating IL-10 *vs* IL-12 is elevated, and the numbers of N2 that express IL-10^hi^ and IL-12^lo^ are increased.^42-43^ Sepsis is dramatically reduced by polarization toward the N1 phenotype with flagellin, a TLR5 agonist.^43^ N1 cells are similarly protective against *S. aureus* infections ^44^ and in inflammatory brain injury.^45^ Additional studies are needed to define the neutrophil subset associated with protection *vs* susceptibility in this model, along with a possible role for other cell types. Finally, the ability to modulate infection resistance using sex hormone modulation should be further explored to better prevent hese potentially lethal infections.

## Figures and Tables

**Table 1:** Frequency of dentoalveolar abscesses and sepsis in anti-IL-1-treated mice

	Treatment		
Sex	Anti-IL-1α	Anti-IL-1β	Anti-IL-1a/β
Male	0/8	0/8	6/8^*^
Female	0/8	0/8	1/8

IL, interleukin.

* *P*<0.05 *vs* females.

**Table 2:** Effect of estrogen modulation on abscesses and sepsis in anti-IL-1-treated female mice

Treatment	Control IgG	Anti-IL-1α/β
Sham OVX (control)	0/11^*^	1/11
OVX	2/11	7/10
OVX+E2 implant	0/11	2/11^†^

IgG, immunoglobulin G; IL, interleukin; OVX, ovariectomy.

* Frequency of abscesses/sepsis.

† *P*<0.01 by *χ*^2^.
